# Feasibility and Acceptability of Objective Pain Assessment Using Multimodal Sensing Signals in Older Adults with Alzheimer's Disease: Preliminary Results of a Pilot Study

**DOI:** 10.1002/alz.088231

**Published:** 2025-01-09

**Authors:** Fernanda Souza Andrade, Julie Ornelas, JuYoung Park, Gabriella Engstrom, Richard D. Shih, Ilknur Telkes

**Affiliations:** ^1^ Florida Atlantic University, Boca Raton, FL USA; ^2^ University of Arizona, Tucson, AZ USA

## Abstract

**Background:**

Chronic pain remains mostly untreated in those with Alzheimer’s disease and related dementias (ADRD), mainly due to limited capacity to verbalize pain. Development of reliable objective biomarkers of chronic pain could improve pain assessment and treatment. We explored feasibility and acceptability of using a wearable electroencephalograph (EEG) and a screen‐based eye tracker system to identify neural signatures of chronic pain in this population.

**Method:**

Inclusion criteria were ADRD in an early to moderate stage defined by Mini‐Montreal Cognitive Assessment (Mini‐Moca) and Quick Dementia Rating System (QDRS) scores and chronic pain (3 months or longer). Participants underwent a single‐session test where participant‐reported outcome (PRO) measures were obtained with 32‐channel EEG. Neural signals were recorded at resting and during a cognitive task with visual stimuli. Attention to the stimuli was captured using an eye‐tracking system.

**Result:**

Five of 29 applicants met all criteria and were included. The majority were excluded due to dementia severity or lack of chronic pain. Four subjects (3 females) concluded the study. Participants' ages ranged from 76 to 93 years. They were diagnosed with Alzheimer’s disease, dementia of unspecified type, or dementia related to Parkinson’s disease. Mini‐Moca scores ranged from 2‐8 of 15 and QRDS ranged from 6‐20 of 30. On Visual Analog Scale for pain, three participants rated mild to moderate pain (2 to 5 out of 10) and one rated severe pain (9 out of 10). Three reported that pain significantly affected their ability to walk, relationships with people, and enjoyment of life. All participants reported feeling comfortable with the EEG cap and eye tracker, were satisfied with the study, and would recommend the study to others. In terms of feasibility, the major challenge was to calibrate the eye tracker system and the duration of the cognitive task which was 30 minutes.

**Conclusion:**

While using a table‐top eye tracker was comfortable for all participants, the calibration phase was difficult. In one case, the image‐based task was not feasible which might be related to participant having moderate dementia. Our preliminary work suggests that wearable modalities may work better in people with ADRD and carry the potential to assess pain objectively.

